# Properties of Flavonoids Isolated from the Bark of *Eysenhardtia polystachya* and Their Effect on Oxidative Stress in Streptozotocin-Induced Diabetes Mellitus in Mice

**DOI:** 10.1155/2016/9156510

**Published:** 2016-09-07

**Authors:** Rosa Martha Perez-Gutierrez, Abraham Heriberto Garcia-Campoy, Alethia Muñiz-Ramirez

**Affiliations:** ^1^Laboratory of Natural Products Research, School of Chemical Engineering and Extractive Industries, National Polytechnic Institute, Unidad Profesional Adolfo Lopez Mateos, Av. Instituto Politecnico Nacional S/N, 07708 Ciudad de Mexico, Mexico; ^2^Potosine Institute and Scientific and Technological Research (CONACYT), A.C. Dam Road, San Jose 2055, Lomas 4A Section, 78216 San Luis Potosi, SLP, Mexico

## Abstract

Six new flavonoids 2′,4′-dihydroxychalcone-6′-O-*β*-d-glucopyranoside (**1**), *α*,3,2′,4′-tetrahydroxy-4-methoxy-dihydrochalcone-3′-C-*β*-glucopyranosy-6′-O-*β*-d-glucopyranoside (**2**), 7-hydroxy-5,8′-dimethoxy-6′*α*-l-rhamnopyranosyl-8-(3-phenyl-trans-acryloyl)-1-benzopyran-2-one (**3**), 6′7-dihydroxy-5,8-dimethoxy-8(3-phenyl-trans-acryloyl)-1-benzopyran-2-one (**4**), 9-hydroxy-3,8-dimethoxy-4-prenylpterocarpan (**5**), and *α*,4,4′-trihydroxydihydrochalcone-2′-O-*β*-d-glucopyranoside (**6**) were isolated from bark of* Eysenhardtia polystachya.* Antidiabetic activity of compounds** 1**–**5** in terms of their cellular antioxidant and free radical scavenging and also in streptozotocin- (STZ-) induced diabetic mice was evaluated on liver transaminases, lipid peroxidation, total bilirubin, total protein, superoxide dismutase (SOD), catalase (CAT), glutathione peroxidase (CSH-Px), and glutathione reductase (GSH). Results indicated that** 1**–**5** scavenged 2,2-diphenyl-1-picrylhydrazyl (DPPH), hydroxyl (^∙^OH), nitric oxide radicals (NO^∙^), superoxide anion radical (O_2_^∙−^), radical cation (ABTS^∙+^), and hydrogen peroxide (H_2_O_2_) radical, and protection against H_2_O_2_ induced BSA damage was also observed. Furthermore,** 1**–**5** showed ability to decrease the oxidative stress in H9c2 cell. Diabetic mice present high levels of lipid peroxide, total protein, SGPT, SGOT, ALP, and TB. However, treatment of STZ-induced diabetes in mice with** 1**–**5** reduced levels of these enzymes leading to protector effect of liver. In addition, with treatment with** 1**–**5**, increases in radical scavenging enzymes of CSH-Px, SOD, GSH, and CAT have also been observed in diabetic mice. The antioxidant properties of compounds** 1**–**5** are a promising strategy for ameliorating therapeutic effects by avoiding disorders in the normal redox reactions in healthy cells which consequently could alleviate complications of diabetes.

## 1. Introduction

Oxidative stress is a consequence of the increased reactive oxygen species (ROS) production and/or a decrease in their elimination. ROS such as hydrogen peroxide (H_2_O_2_), superoxide anion (O_2_^∙−^), hydroxyl radical (OH), nitrogen oxide (NO), and lipid peroxides are formed in aerobic metabolism as normal products but also are produced under pathophysiological conditions in elevated rates. ROS can be responsible for the attack to biological macromolecules such as nucleic acids, proteins, membrane lipids, and carbohydrates, causing damage in the cell, which has been implicated in cardiovascular diseases, cancer, neurodegenerative disorders, and diabetes [1]. Natural antioxidants present in the majority of plants help in preventing mutagenesis, aging, and carcinogenesis and reduce oxidative damage due to their radical scavenging effect [2].

The tree* Eysenhardtia polystachya* (Ortega) Sarg, belonging to the Leguminosae family, is known as “palo azul” and has widely been used as antirheumatic, for the treatment of nephrolithiasis and bladder disorders developed in diabetes [3]. Phytochemical studies indicate that* E. polystachya* contains polyphenols [4]. In another study, isoflavones displayed moderate cytotoxic activity against KB cell lines [5]. In previous studies, methanol-water extract from the bark of* E. polystachya* was evaluated in* in vitro* assays, showing antioxidant potential, hypoglycemic, and AGEs inhibition capacity [6]. In this study, we investigated the antioxidant properties and protective activity of 5 flavonoids isolates from the bark of* E. polystachya* in* in vitro* and* in vivo* assays.

## 2. Materials and Methods

### 2.1. General Experimental Procedures

IR spectra were determined on a Perkin-Elmer 1720 FTIR. ^l^H-^l^H, ^l3^C, DEPT, ID, DQF-COSY, TOCSY, and HMBC experiments were recorded on a Bruker DRX-300 NMR spectrometer, operating at 599.19 MHz for ^1^H and 150.86 MHz for ^l3^C, using the UXNMR software package; chemical shifts are expressed in *δ* (ppm) using TMS as an internal standard [7]. HREIMS were recorded on a JEOL HX 110 mass spectrometer (JEOL, Tokyo, Japan). TLC and column chromatography were carried out using precoated TLC silica gel 60 F254 aluminium sheets from Sigma-Aldrich (St. Louis, USA) and silica gel 60 (230–400 mesh, Merck Co., New Jersey, USA); solvents used as eluents were purchased from Fermont (California, USA). DCFH-DA were purchased from Degussa (Cincinnati, USA). All other reagents were obtained from Sigma-Aldrich (St. Louis, USA).

### 2.2. Plant Material

Bark of* E. polystachya* was collected in October 2014 near Tula, State of Mexico. A voucher specimen (number 49584) is deposited at the Herbarium of Universidad Autonoma Metropolitana-Xochimilco, Mexico.

### 2.3. Extraction and Isolation

The dried bark* E. polystachya* (5 kg) was milled and macerated with distilled water and methanol (1 : 1) for 15 days. The extract was evaporated under reduced pressure at 40°C, affording 400 g which was partitioned sequentially with n-hexane, CHCl_3_, and MeOH. The methanol extract was adsorbed on 50 g of silica gel 60 (70−230-mesh ASTM), loaded onto a column of silica gel of 50 cm length and 7 cm diameter, 300 g silica gel, and then eluted with CH_2_Cl_2_/EtOAc 9 : 2 to yield 9 subfractions (PA-1 to PA-9). The elutions were examined by TLC and each one of them was determined as antioxidant activity. The fractions having antioxidant activity were PA-5 and PA-7. PA-5 was subjected to silica gel column chromatography eluted with EtOAc/hexane 7 : 2 to yield 7 subfractions (PA5-1 to PA5-7). Subfractions PA5-2 and PA5-7 were subjected to gel filtration over Sephadex LH-20 eluted with CHCl_3_/MeOH (6 : 1), and this led to the isolation of compounds** 7**–**13** which were combined and further purified by repeated preparative thin layer chromatography (PTLC) eluting with ethyl ether/acetone/methanol (1.5/3/0.5) gradient system. This led to the isolation of compounds** 7** (65.0 mg),** 8** (13.5 mg),** 9** (76.4 mg),** 10** (15.8 mg),** 11** (49.2 mg),** 12** (29.37 mg), and** 13** (48.5 mg). PA-7 was subjected to silica gel column chromatography eluted with EtOAc/hexane 9 : 3 to yield 6 subfractions (PA7-1 to PA7-6). Fractions PA7-4 and PA7-5 were combined and further crude mixture was subjected to preparative thin layer chromatography (PTLC) eluting with ethyl ether/acetone/methanol (4/2/1.0) gradient system. Subfractions 1–3 were purified by gel filtration over Sephadex LH-20 eluted with CH_2_Cl_2_-CH_3_OH 1 : 1 increasing concentration of MeOH yielding compounds** 1** (67.1 mg),** 2** (85.4 mg),** 3** (72.8 mg),** 4** (96.7 mg),** 5** (90.2 mg), and** 6** (42.9 mg).

### 2.4. Antioxidant Activity* In Vitro*

#### 2.4.1. Scavenging Effects on DPPH Radicals

 1 mL solution of 2,2-diphenyl-1-picrylhydrazyl‎ (DPPH, 0.2 mmol/L) in methanol was added to 4 mL of sample. The mixture was shaken vigorously and was allowed to stand at room temperature for 30 min. Absorbance of the resulting solution was measured at 517 nm using a spectrophotometer (UV-1800, Shimadzu, Kyoto, Japan) [8].

### 2.5. Chelating Activity on Metal Ions

40 *μ*mol/L FeCl_2_ was reacted with flavonoids for 5 min. Then ferrozine (200 *μ*mol/L) was added and the mixture was left to stand for another 10 min. The absorbance at 562 nm was determined spectrophotometrically (UV-1800, Shimadzu, Kyoto, Japan) [8].

### 2.6. Trolox Equivalent Antioxidant Capacity (TEAC) Assay

 2 mmol/L H_2_O_2_ alone in 30 mmol/L acetate buffer with pH 3.6 was incubated for 30 min at room temperature producing 10 mmol/L of ABTS radical cation (ABTS^∙+^). 80 *μ*L of this solution was added to 0.4 mol/L acetate buffer (pH 5.8, 800 *μ*L) and to 20 *μ*L of the sample solutions; after exactly 5 min the absorbance was read at 734 nm. A dose-response curve was plotted for Trolox and antioxidant ability was expressed as TEAC value [9].

### 2.7. Scavenging Effects on Nitrite Oxide (NO)

In the test tubes to 0.1 mL of sodium nitroprusside solution (25 mmol/L) was added 150 *μ*g/mL of the sample; then the tubes were incubated for 150 min at room temperature. Griess reagent (0.3 mL of 1 g/L naphthylethyl-enediamine dihydrochloride and 0.3 mL of 10 g/L sulfanilamide in 5% H_3_PO_4_) was added to the incubation solution. The absorbance was immediately read at 570 nm and referred to the absorbance of standard solutions of sodium nitrite salt treated in the same way with Griess reagent [10].

### 2.8. Effects on the Oxidation of BSA Induced by H_2_O_2_

 0.2 mL of samples was incubated with 1 mL BSA (40 mg/mL), 0.4 mL H_2_O_2_ (20 mmol/L), and 0.4 mL of 20 mmol/L phosphate buffer (pH 7.4), for 2 h at 37°C. 5,5-Dithio-bis(2-nitrobenzoic acid) (DTNB, 1 mL, 2 mmol/L) was then added and the mixture was left to stand for another 30 min. The absorbance was measured at 410 nm. The free thiol concentration of samples was calculated based on the standard curve prepared by using various concentrations of l-cysteine.

### 2.9. Superoxide Radical Scavenging Activity (O_2_^∙−^)

The reaction mixture is carried out in final volume of 3 mL containing 6 mM EDTA, 20 *μ*M riboflavin, 58 mM phosphate buffer at pH 7.6, and 50 *μ*M of nitroblue tetrazolium chloride (NBT). The reaction mixture is exposed for 15 min to 40 V under fluorescence lamp to initiate the reaction. The absorbance was determined at 560 nm [11].

### 2.10. Hydroxyl Radical Scavenging Activity

The reaction mixture contained phosphate buffer (0.1 mM, pH 7.4), 2-deoxy-2-ribose (2.8 mM), ferric chloride (20 *μ*M), hydrogen peroxide (500 *μ*M), ascorbic acid (100 *μ*M), and EDTA (100 *μ*M) and test sample (10–1000 *μ*g/mL^−1^) in a final volume of 1 mL. The mixture was incubated at 37°C for 1 h. Then 0.8 mL of the mixture was added to 2.8% of trichloroacetic acid (TCA) solution (1.5 mL), followed by sodium dodecyl sulphate (0.2 mL) and a thiobarbituric acid (TBA) solution (1 mL of 50 mM at 1% in sodium hydroxide). The mixture was heated (90°C for 20 min) to develop the colour. After cooling, the absorbance was determined at 532 nm [12].

### 2.11. Hydrogen Peroxide Radical Scavenging Activity

Compounds at concentrations of 1 mg/mL were dissolved in 3.4 mL of a 0.1 M phosphate buffer (pH 7.4) solution and then were mixed with 600 *μ*L of a 43 mM solution of H_2_O_2_ prepared in the same buffer and their concentration was measured by reading absorbance values at 230 nm of the reaction mixtures [13].

### 2.12. Cellular Oxidative Stress Inhibition

The intracellular accumulation of ROS in the H9c2 rat cardiomyoblast cell line was determined with carboxy-2′,7′-dichloro-dihydro-fluorescein diacetate (DCFHDA) method [14]. Cells H9c2 rat heart-derived embryonic myocytes are obtained from American Type Culture Collection, Manassas, VA, USA (CRL-1446), which was cultured with DMEM/F12 supplemented with 100 U/mL penicillin G, 10% (v/v) foetal bovine serum, 2 mM/L-glutamine, and 100 mg/mL streptomycin. Cells were incubated using 5%  CO_2_ and 95% air at 37°C, after the cells were plated in 35-mm culture dishes at 5.0 × 10^−4^ cells/cm^2^. Then, the medium is changed with a new one. Cells seeded on 96-well plates were incubated with DCFHDA probe for 40 min. At the end of this period, medium was removed and cells were exposed to the flavonoids under investigation at a concentration of 10, 50, and 100 *μ*g/mL. After incubating exposed cells at 37°C for 24 h, fluorescence was measured at 488 nm (excitation) and 535 nm (emission) wavelengths on a microplate reader (Molecular Devices Spectra MAX Gemini X).

### 2.13. Evaluation of Oxidative Stress Markers* In Vivo*

#### 2.13.1. Animals Care Conditions

The study was conducted in male CD1 mice, weighing about 25–30 g. Before and during the experiment, animals were fed a standard laboratory diet (Mouse Chow 5015, Purina) with free access to water. Mice were procured from the bioterium of ENCB and were housed in microloan boxes in a controlled environment (temperature 25 ± 2°C). Animals were acclimatized for a period of three days in their new environment before the initiation of experiment. Litter in cages was renewed three times a week to ensure hygiene and maximum comfort for animals. The experiments reported in this study were following the guidelines stated in Principles of Laboratory Animal Care (NIH publication 85-23, revised 1985) and the Mexican Official Normativity (NOM-062-Z00-1999). All animals procedures were performed in accordance with the recommendations for the care and use of laboratory animals (756/lab/ENCB).

### 2.14. Induction of Mild Diabetes (MD)

Mild diabetes type 2 was induced in overnight fasted mice by a single intraperitoneal injection of 45 mg/kg streptozotocin (STZ) dissolved in 0.1 mol/L cold citrate buffer (pH 4.5), 15 min after the intraperitoneal administration of 120 mg/kg nicotinamide. The STZ treated animals were allowed to drink 5% glucose solution overnight to overcome drug induced hypoglycemia. After 7 days of development of diabetes, mice with moderate diabetes having persistent hyperglycaemia with more than 200 mg/dL were used for further experimentation [15].

### 2.15. Experimental Design

Eight groups (*n* = 10) of diabetic mice were used to determine the chronic effect of** 1**–**5**. Each group was submitted to a specific treatment, as follows. Normal control and mild diabetic rats groups were fed with normal diet and drinking water* ad libitum* and were given saline by gastric gavage Mice with mild diabetes daily treated with the compounds isolated by an oral gavage at doses of 20 mg/kg of weight. The flavonoids were dissolved in distilled water and administered orally as a daily dose for four weeks. Mice were fasted overnight and sacrificed by cervical dislocation. Blood samples were collected in EDTA coated tubes. Livers, kidney, and pancreas were removed, washed with cold saline, and stored at −80°C.

### 2.16. Antioxidant Parameters Levels in Serum, Liver, Pancreas, and Kidney

Activities of superoxide dismutase (SOD), serum catalase (CAT), glutathione peroxidase (CSH-Px), and glutathione reductase (GSH) were measured by assay kits purchased from Cayman Chemical (Michigan, USA), and the procedures were according to the kits instructions. The protein concentration was determined by the Bradford method [16] as described in the Bio-Rad protein assay kit. Lipid peroxidation (LPO) is used as an indicator of the oxidative stress in tissues and was estimated by the method of Fraga et al. [17], expressed as *μ*M/g of liver and kidney tissue. Malondialdehyde (MDA) as thiobarbituric acid reactive substance was measured at 532 nm spectrophotometrically [18]. Serum glutamate oxaloacetate transaminase (SGOT), glutamate pyruvate transaminase (SGPT), serum alkaline phosphatase (SALP), total bilirubin (TB), and total protein were determined by using a commercial Diagnostic Kit Biocompare, BioVision, Biocompare, and Thermo Scientific, respectively.

## 3. Results and Discussion

### 3.1. Characterization of the Isolated Compounds

**Figure 1:**
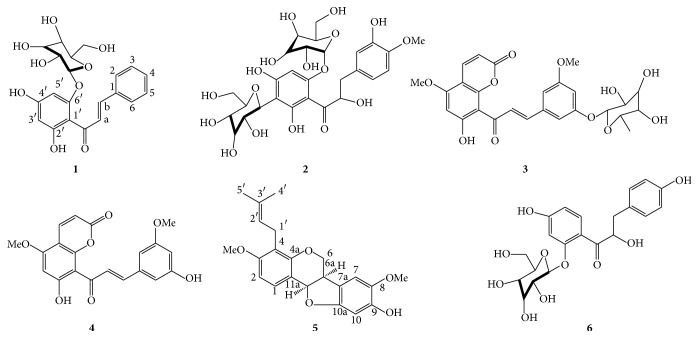
New compounds isolated from* Eysenhardtia polystachya*.

The MeOH extract was subjected to multiple chromatographic purifications to afford dihydrochalcones** 1**–**13** (Figures 1 and 3). The spectral data of the six new compounds isolated (**1**–**6**) are presented as follows.

2′,4′-Dihydroxychalcone-6′-O-*β*-d-glucopyranoside (**1**) was isolated as dark yellow amorphous powder, mp 138–140°C; UV *λ*_max_ 362, 301, and 238 nm; IR (KBr) cm^−1^: 3341, 2957, 2761, 1611, 1597, 1506, and 1463; and HRESIMS* m/z* 418.1270 calc. For C_21_H_22_O_9_, 418.1264, and ^1^H and ^13^C NMR data see Table 1.

**Figure 2:**
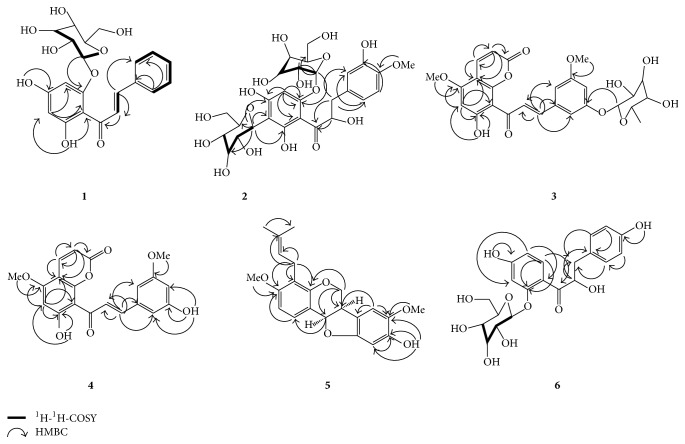
Correlation of ^1^H-^1^H-COSY and HMBC compounds** 1**–**6**.

Compound** 1** was isolated from ethanol-water 1 : 1 extract of bark of* Eysenhardtia polystachya* after purification through silica gel column chromatography. Compound** 1** showed a molecular ion at* m/z* 418.1270 in its HREIMS and the presence of 21 carbons on the ^13^C NMR spectrum suggested a molecular formula C_21_H_22_O_9_. DEPT experiments classified the protonated carbon signals into one methylene, fourteen methines, and six quaternary carbons. The IR spectrum of** 1** showed absorption bands at *ν*_max_ 3341, 2957, 2761, 1597, 1506, 1463, and 1611 cm^−1^ showing the presence of hydroxy, aromatic, and carbonyl groups. The ^1^H NMR spectrum showed the presence of unsubstituted ring B (*δ*_H_ 7.12, 2H, m, H-2, 6, 7.46, 3H, m, H-3, 4, 5), and it showed a pair of doublets of *δ*_H_ 8.24 (H-*β*) and *δ*_H_ 7.50 (H-*α*) with the vicinal coupling constant ^3^ *J* = 15.8 Hz due to* trans*-*α*,*β*-unsaturated ketone protons. Its ketone carbonyl was confirmed with the signal at *δ*_C_ 202.69, C-*α* (*δ*_C_ 141.31), and C-*β* (*δ*_C_ 130.60) in the ^13^C NMR being indicative of chalcone skeleton. NMR spectrum showed signals for one chelated hydroxyl group *δ*_H_ 13.2 (1H, s, OH-2′) and one singlet at *δ*_H_ 6.81 assigned to OH-4′. In addition ^1^H NMR spectrum showed the presence of two aromatic protons in ring A (Table 1). These were placed at C-3′ and C-5′ based on correlation of NOESY and HMBC spectrum (Figure 2). In the HMBC spectrum the long range correlation of H-3′ with C-1′ (*δ*_C_ 103.79) and C-4′ (*δ*_C_ 148.36), H-5′ with C-1′ (*δ*_C_ 103.79), C-3′ (*δ*_C_ 114.82), C-4′ (*δ*_C_ 148.36), and C-6′ (*δ*_C_ 161.21), and H-1′′ with C-6′ (*δ*_C_ 130.60), C-2′′ (*δ*_C_ 78.36), and C-5′′ (*δ*_C_ 71.17) was observed. Thus, the structure of compound** 1** was established as 2′,4′-dihydroxychalcone-6′-O-*β*-d-glucopyranoside (Figure 1).

*α*,3,2′,4′-Tetrahydroxy-4-methoxy-dihydrochalcone-3′-C-*β*-glucopyranosy-6′-O-*β-* d *-*glucopyranoside (**2**) was isolated as dark yellow amorphous powder, mp 150–152°C; UV *λ*_max_ 235, 264, 302, and 312 nm; IR (KBr) cm^−1^: 3339, 1621, 1598, 1510, and 1460; and HRESIMS* m/z* 644.1936 calc. For C_28_H_36_O_17_, 644.1952, and ^1^H and ^13^C NMR data see Table 1.

Compound** 2** was also isolated as dark yellow amorphous powder. The aliphatic hydroxyl group of compound** 3** has *α* position similarly to** 2**. The singlet at *δ*_H_ 3.85 was assigned to an aromatic methoxyl group which showed long range correlation with C-4 (*δ*_C_ 145.05) in the HMBC spectrum indicated to be linked at position C-4, whereas the doublets at *δ*_H_ 4.85 and *δ*_H_ 5.01 were attributed to two *β*-glucopyranose moieties on the basis of the coupling constant (7.5 and 7.3 Hz). In the HMBC spectrum of** 2**, the long range correlations of anomeric proton H-1′′ (*δ*_H_ 4.85) with C-2′ (*δ*_c_ 164.13), C-3′ (*δ*_c_ 107.88), C-4′ (*δ*_c_ 165.34), C-2′′ (*δ*_c_ 74.73), C-3′′ (*δ*_c_ 78.85), and C-5′′ (*δ*_c_ 77.32) were observed. These signals suggested that glucose unit should be directly linked at position C-3′. Also the anomeric proton H-1′′′ (*δ*_H_ 5.01) correlation with C-6′ (*δ*_c_ 164.04), C-2′′′ (*δ*_*δ*_C__ 78.65), and C-5′′′ (*δ*_c_ 78.01) indicated that the other glucose unit should be linked to C-6′ through a glycosidic bond (Table 1). ^1^H NMR spectrum showed the typical pattern of a 2′,3′,4′,6′-tetrasubstituted and 3,4-disubstituted chalcone with a singlet for one proton at *δ*_H_ 6.68 could be assigned to the C-5′ indicating that this position is free. Analysis of the HMBC and COSY spectra of** 3** allowed for the complete assignment of all protons and carbons (Figure 2). Consequently, the structure of compound** 3** was elucidated as *α*,3,2′,4′-tetrahydroxy-4-methoxy-dihydrochalcone-3′-C-*β*-glucopyranosy-6′-O-*β*-d-glucopyranoside (Figure 1).

7-Hydroxy-5,8′-dimethoxy-6′*α*-l-rhamnopyranosyl-8-(3-phenyl-trans-acryloyl)-1-benzopyran-2-one (**3**) was isolated as dark yellow amorphous powder, mp 142–144°C; UV *λ*_max_ 249 and 381 nm; IR (KBr) cm^−1^: 3439, 1659, 1619, 1521, and 1443; HRESIMS* m/z* 514.1483 calc. For C_26_H_26_O_11_, 514.1475, and ^1^H and ^13^C NMR data see Table 1.

Compound** 3** was isolated as dark yellow amorphous powder. Its molecular formula was assigned as C_26_H_26_O_11_ on the basis of HRESIMS and ^13^C NMR data. The IR spectrum showed characteristic signals for OH (3439 cm^−1^), aromatic ring (1521 and 1443 cm^−1^), and *α*,*β*-unsaturated ketone (1659 and 1619 cm^−1^). The UV spectrum at 249 and 381 nm confirmed the existence of unsaturated functional groups. The ^1^H NMR spectrum showed one single proton at *δ*_H_ 14.15 (1H, s, 7-OH) and two methoxyl groups at *δ*_H_ 3.39 (3H, s) and *δ*_H_ 3.46 (3H, s), while the resonances of two trans-coupled olefinic protons were recorded at *δ*_H_ 7.32 (1H, d, *J* = 15.4 Hz, H-2′) and 7.32 (1H, d, *J* = 15.4 Hz, H-3′) as well as two olefinic protons *δ*_H_ 6.58 (1H, d, *J* = 10.1 Hz, H-3) and *δ*_H_ 7.50 (1H, d, *J* = 10.1 Hz, H-4). In addition ^1^H NMR spectrum showed aromatic signals of 4H at 6.32–6.47 and also exhibited signals due to *α*-l-rhamnopyranosyl at *δ*_H_ 5.05 (1H, brs, H-1′′) and *δ*_H_ 1.15 (3H, d, 7.0 Hz, H-6′′). ^13^C NMR and DEPT spectra (Table 1) indicated signals corresponding to 26 carbons which were classified into three CH_3_ groups, thirteen groups, and ten quaternary carbons. The attachment of the rhamnopyranosyl moiety was deduced to be at C-6′, by the HMBC spectrum in which the anomeric proton of the rhamnopyranosyl moiety at *δ*_H_ 5.05 (1H, brs, H-1′′) showed long range correlation with C-6′ at *δ*_C_ 163.56. ^13^C NMR data, with the corresponding literature, suggested that** 3** exhibited the skeletons of a neoflavone and a chalcone [19, 20]. The HBMC correlation of H-3 at *δ*_H_ 6.58 with C-2 (*δ*_C_ 168.21) and C-4a (*δ*_C_ 105.97) and H-4 with C-3 (*δ*_C_ 143.48) revealed the existence of the neoflavone skeleton. The HMBC relationship of the double bond group (*δ*_H_ 7.97 and *δ*_H_ 7.70) indicated a fragment of O@CAC@CAC, showing the skeleton of a chalcone. Compound** 3** possesses a methoxyl group at C-5 suggested by a ^13^C-^1^H long range coupling from OCH_3_-5 (*δ*_H_ 3.39) to C-5 (*δ*_C_ 143.48) and another methoxyl group in C-8′ indicated by correlations observed in OCH_3_-8′ (*δ*_H_ 3.46) to C-8′ (*δ*_C_ 166.12) in the HMBC spectrum (Figure 2). This was confirmed by NOE correlation between OMe-5 to H-6 and OMe-8′ with H-9′. On the basis of these data the structure is proposed as 7-hydroxy-5,8′-dimethoxy-6′*α*-l-rhamnopyranosyl-8-(3-phenyl-trans-acryloyl)-1-benzopyran-2-one (Figure 1).

6′7-Dihydroxy-5,8-dimethoxy-8(3-phenyl-trans-acryloyl)-1-benzopyran-2-one (**4**) was isolated as yellow amorphous powder, mp 156–158°C; UV *λ*_max_ 228 and 349 nm; and HRESIMS* m/z* 396.0863 (calcd C_20_H_16_O_7_). For ^1^H and ^13^C NMR data, see Table 2.

The NMR spectra of compound** 4** showed similarities to those of** 3** indicating a close structure. The C-6′ rhamnopyranosyl moiety of** 3** is replaced with a hydroxyl group at *δ*_*C*_ 168.38 as revealed by the spectra of HMBC and COSY showing the corresponding correlations (Figure 2). On the basis of these data the structure is proposed as 6′7-dihydroxy-5,8-dimethoxy-8(3-phenyl-trans-acryloyl)-1-benzopyran-2-one (Figure 1).

9-Hydroxy-3,8-dimethoxy-4-prenylpterocarpan (**5**) was isolated as dark yellow amorphous powder, mp 142–134°C; UV *λ*_max_ 212, 292, and 344 nm; IR (KBr) cm^−1^: 3389, 1619, 1547, 1483, and 1429; and HRESIMS* m/z* 368.1641 calc. For C_22_H_24_O_5_, 368.1624, and ^1^H and ^13^C NMR data see Table 2.

Compound** 5** was isolated as yellow amorphous powder. Its molecular formula was assigned as C_22_H_24_O_5_ on the basis of HRESIMS and ^13^C NMR data. In IR spectrum were observed absorption bands for hydroxyl groups at 3389 cm^−1^ and aromatic groups at 1619, 1547, 1483, and 1429 cm^−1^. Its molecular formula was established by HRESIMS and ^13^C NMR data as C_22_H_24_O_5_. ^13^C NMR and DEPT spectrum indicated that it contains 22 carbons, including four methyls, two methylenes, seven methines, and nine quaternary carbons. A pterocarpan structure was indicated to ^1^H MR spectrum due to the splitting pattern of the protons at *δ*_H_ 4.31 (dd, *J* = 4.9, 10.2 Hz, H-6*β*), *δ*_H_ 3.59, (t, *J* = 10.2, H-6*α*), *δ*_H_ 3.50 (m, H-6a), and *δ*_H_ 5.20 (d, *J* = 7.2, 11a) related to the protons of the heterocyclic ring B. The HMQC spectrum suggested the presence of two OCH_3_ groups at *δ*_H_ 3.11 (3H, s, OMe-3) and *δ*_H_ 3.35 (3H, s, OMe-8), confirming its presence in the ^13^C NMR with signals to *δ*_*C*_ 55.57 (OMe-3) and *δ*_*C*_ 60.64 (OMe-8). HMBC spectrum shows the relations between 3.11 (3H, s, OMe-3) and *δ*_*C*_ 147.76 (s, C-3) and between 3.77 (3H, s, OMe-8) and *δ*_*C*_ 152.51 (s, C-8) show two -OMe that joined with C-3 and C-8 and the relations between OH *δ*_H_ 8.12 (br, s, HO-) and *δ*_*C*_ 99.01 (C-10) and between *δ*_*C*_ 163.17 (s, C-9) and *δ*_*C*_ 152.51 (s, C-8) indicate that hydroxyl group is at C-9. The proton doublets at *δ*_H_ 7.15 and *δ*_H_ 6.52 suggested* ortho*-coupled position. The correlation of H-1′ (*δ*_H_ 3.43) with C-4 (*δ*_*C*_ 115.84), C-4a (*δ*_*C*_ 154.59), and C-3 (*δ*_*C*_ 147.76) in HMBC spectrum indicated that prenyl group was located at C-4 of A ring. The HMBC spectra (Figure 2) show correlation of H-11a (*δ*_H_ 5.20) with C-1 (*δ*_*C*_ 77.75), C-6a (*δ*_*C*_ 40.48), and C-11b (*δ*_*C*_ 77.75) and H-6a (*δ*_H_ 3.50) with C-6a (*δ*_*C*_ 69.37), C-11a (*δ*_*C*_ 77.75), and C-6b (*δ*_*C*_ 116.56) (Figure 2). NOESY correlation of the 11a proton (*δ*_H_ 5.20) with H-1 (*δ*_H_ 7.15) suggested that H-11a is equatorially oriented* cis-*ring fusion between rings B and C. The cisconfiguration of 6a and 11a was confirmed based on the evidence from the chemical shift and* J* value (7.0 Hz) compared with values reported in the literature for cis (6.6 Hz) and trans (13.4) [21]. Key correlations in** 5** are shown in Figure 2. Therefore, the structure of compound** 6** is 9-hydroxy-3,8-dimethoxy-4-prenylpterocarpan (Figure 1).

*α*,4,4′-Trihydroxydihydrochalcone-2′-O-*β*-d-glucopyranoside (**6**) was isolated as dark yellow amorphous powder, mp 146-147°C; UV *λ*_max_ 263, 293, and 328 nm; IR (KBr) cm^−1^: 3341, 2957, 2761, 1611, 1597, 1506, and 1463; and HRESIMS* m/z* 418.1270 calc. For C_21_H_22_O_9_, 418.1264, and ^1^H and ^13^C NMR data see Table 1.

Compound** 6** was isolated as dark yellow amorphous powder. The positive HREIMS analysis showed an ion peak at* m/z* 436.1374 which corresponded to the molecular formula C_21_H_24_O_10_. The IR showed absorption of hydroxyl (3341 cm^−1^) and aromatic rings (1647 and 1489 cm^−1^) functions. Aliphatic proton was assigned to ABX system at *δ*_H_ 4.70 (1H, d, 7.5 Hz, H-*α*), *δ*_H_ 2.99 (1H, 16.7, 3.0 Hz, H-*β*), and *δ*_H_ 2.81 (1H, 16.7, 3.2 Hz, H-*β*) suggesting the presence of -CO-CH (OH)-CH2– moiety. Compound** 6** showed the presence of a glucopyranosyl moiety which was linked to C-3′ according to the HSQC correlation of anomeric proton at *δ*_H_ 5.02 (br, d, *J* = 7.3 Hz, H-1′′) with C-3′ (*δ*_*C*_ 164.03). The anomeric configuration was assigned as *β* for the glucopyranosyl group from their coupling constants. The downfield shift of C-4′ at *δ*_*C*_ 144.59 was indicative that it has a hydroxy group. Aromatic protons H-3′ (*δ*_H_ 6.68), H-5′ (*δ*_H_ 6.44), and H-6′ (*δ*_H_ 7.71) suggested a 2′,4′-dioxygenation in a trisubstituted ring, which is corroborated by the NOESY and the HMBC spectrum (Figure 2). The AA′XX′ spin system (*δ*_H_ 6.71, 7.22) of A ring indicates 1,4-disubstitution, whereas the chemical shift of C-4 (*δ*_*C*_ 164.03) reveals hydroxyl group at this position. Therefore, the structure of compound** 6** was identified as *α*,4,4′-trihydroxydihydrochalcone-2′-O-*β*-d-glucopyranoside (Figure 1).

**Figure 3:**
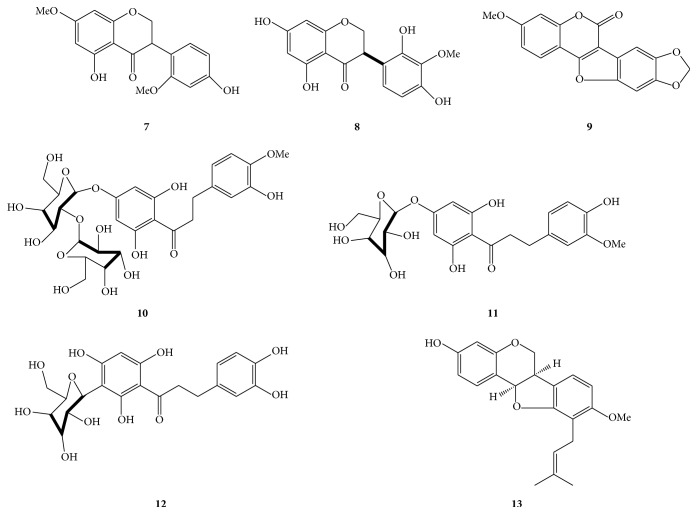
Known compounds isolated from* Eysenhardtia polystachya*.

In addition, seven known compounds were also isolated (Figure 3) by comparing the spectroscopic data with literature and were identified as 5,4′-dihydroxy-7,2′-dimethoxyl-isoflavone (**7**) [22], (3R)-5,7-2′,4′-tetrahydroxyl-3′-methoxyl-isoflavanone (**8**) [23], flemichapparin C (**9**) [24], neohesperidin dihydrochalcone (**10**) [25], hesperetin dihydrochalcone glucoside (**11**) [26], aspalathin (**12**) [27], and* sandwicensis* (**13**) [28].

### 3.2. Antioxidant Activity

Radical scavenging capacities of compounds** 1**–**5** isolated from* E. polystachya* are given in Table 3. However, compound** 6** displayed lower scavenging value and the data are not presented here. Chalcones** 4**,** 3**,** 5,** and** 1** showed significant scavenging activity in DPPH radical with IC_50_ values of 10.21 to 19.39 *μ*g/mL. These results suggested that these compounds showed the highest hydrogen-donating capacity towards the DPPH radical.

Hydroxyl radicals are found in living systems that react with almost all biomolecules as they are high oxidant. The orders of reactivity of the flavonoids were similar to that of DPPH. Compound** 4** (33.17 *μ*g/mL) was found to exhibit the highest hydroxyl radical scavenging effect followed by** 5** (36.52 *μ*g/mL) and** 3** (37.45 *μ*g/mL). Scavenging of hydrogen peroxide by the isolated compound was found to be better compared to the ascorbic acid (IC_50_ 443.76 *μ*g/mL). Among the tested flavonoids, the best scavenging of hydrogen peroxide activity was presented by** 3** (IC_50_ 80.73 *μ*g/mL),** 4** (IC_50_ 87.68 *μ*g/mL), and** 1** (IC_50_ 91.13 *μ*g/mL). The presence in the –OH group in ring A and -OCH3 in ring B showed good antioxidant activity on scavenging of hydrogen peroxide activity compared to electron-withdrawing substituents at paraposition.

Superoxide radical is a free radical harmful to cellular components, precursor for many reactive oxygen species. Superoxide radical scavenging capacity followed an activity order of chalcones** 4**,** 3,** and** 5**.

As evident from Table 3, the isolated compound exerted nitric oxide scavenging activities with values of IC_50_ of 25.47 to 75.04 *μ*g/mL. Compound** 4** showed the highest scavenging effects on nitric oxide, followed by** 3**,** 5,** and** 1**. Reactive nitrogen species (N_2_O_4_, NO_3_, N_3_O_4_, NO, and NO_2_) are formed during the reaction with superoxides or oxygen which are responsible for altering the functional and structural cellular components.

The ability to chelate and deactivate transition metals is an important mechanism of antioxidant activity, which catalyzes Fenton-type reactions and hydroperoxide decomposition. Therefore, it was considered of importance to study chelating ability of the isolated compound with the metal. The chelating activity of flavonoids to suppress the formation of ferrozine-Fe^2+^ complex decreased in the order of** 4** >** 5** >** 3** >** 1** >** 2**.

Chalcones** 4** and** 3** and pterocarpan** 5** also showed good significant scavenging activities of Trolox (16.23 to 20.34 nM) indicating their good antioxidant effect.

The inhibition of the isolated compound on the oxidation of BSA induced by H_2_O_2_ was also determined. Exposure of H_2_O_2_ to BSA produced a decrease of free thiol groups level. Results are shown in Table 3; the isolated compound significantly inhibited the oxidation of BSA induced by H_2_O_2_ in a dose-dependent manner. Among the five compounds tested,** 4** showed the strongest antioxidant activity at a concentration of 300 mg/mL preventing the 97% of the depletion of protein thiol groups. These results indicated that hydrophilic antioxidant moiety in the compounds has the ability to scavenge H_2_O_2_ reducing thiol groups oxidation in BSA.

The antioxidant activity in all tested assays indicates that the compound with chalcone skeleton showed the best inhibitory activity when the dihydrochalcone as in compounds** 4**,** 3,** and** 1** indicated that chalcones having electron donor group in the phenyl ring A in orthoposition to carbonyl moiety showed better antioxidant activity. Moreover, the activity decreasing obviously with glycosidation such as only** 1** showed a moderate inhibitory activity, and the other glycoside** 2** showed a low inhibitory effect. However, pterocarpan showed a good antioxidant activity in various oxidation systems.

Antioxidants compounds have been shown to delay, inhibit, and prevent the oxidation, possibly through the mechanisms interacting with biological systems as scavenging free radicals, absorbing oxygen radicals, and chelating of the metal ions [29].

### 3.3. Flavonoids Prevent Palmitate-Induced ROS Production and Oxidative Stress in H9c2 Cells

The ability of** 1**–**5** to decrease the oxidative stress in cells was determined. It is well known that concentrations of palmitate at 500 *μ*m increase oxidative stress and stimulate ROS production in H9c2 cell. H_2_O_2_ model has been used to determine the oxidative stress because it can generate superoxide anion radical. Pretreatment with flavonoids for 1 hr at 20 *μ*M significantly decreased ROS production preventing oxidative stress in H9c2 cell and palmitate-induced ROS production using DCFH-HA (for H_2_O_2_^−^) and DHE (for O_2_^−^) test. The oxidative stress reduction potential of** 1**–**5** is given in Table 4. The capacity of 10, 50, and 100 *μ*g/mL flavonoids to reduce the oxidative stress was compared with that of 25 *μ*g/mL of ascorbic acid. The values of the mean fluorescent intensity indicated that the isolated compound significantly reduces the increase of ROS in palmitate-induced cells. The antioxidant effectiveness* in vitro* of the isolated compound can be possibly due to their ability to act as free radical scavengers, quenchers of singlet O_2_ formation, to complex with prooxidant metal ions and reducing agents [30].

### 3.4. Effects of Flavonoids on Oxidative Stress Markers and Aminotransferases Activities

There were no changes of blood glucose level after 30 days of administration of** 1**–**5**. There was a significant increase of lipid peroxide in the liver, kidney, and pancreas in diabetes mice. However, the administration of** 1**–**5** improved these levels in the treated groups with respect to the diabetic control group as shown in Table 5. The production of ROS including hydroxyl, superoxide anions and hydrogen peroxide, nitric oxide, DPPH, ABTS radicals, protein oxidation, and lipid peroxidation can be enhanced by treatment with isolated** 1**–**5**, and increases in radical scavenging enzymes of CSH-Px, SOD, GSH, and CAT have also been observed in liver, pancreas, and kidney in the group of diabetic animals treated.

Elevation of serum biomarker enzymes such as total protein, SGPT, SGOT, ALP, and TB was observed in diabetic mice indicating deterioration in liver function (Table 6) which may be because STZ produced liver damage causing leakage of these enzymes into the blood [31]. However, treatment of STZ-induced diabetes in mice with** 1**–**5** reduced levels of these enzymes leading to hepatoprotective effect.

### 3.5. Effects of Flavonoids on TBARS Levels

In diabetic mice, elevated levels of oxidative stress are due to protein glycation, autoxidation of glucose, low activities of antioxidant enzymes, and lipid peroxidation [32]. Consistent with this finding, decreased activities of antioxidant enzymes, such as GSH, SOD, GST, and GPx, and a marked increase in the concentration of TBARS indicate increase lipid peroxidation leading to decrease of the antioxidant defense mechanisms to avoid overproduction of ROS which leads to tissue injury [33]. Supplementation to diabetic mice with the isolated compound resulted in a significant (*p* < 0.05) diminution in lipid peroxidation levels, in liver and kidney compared with diabetic control, and the observed reduction of TBARS towards normal levels (Table 7). These results suggest a marked inhibition of the isolated compound on ROS generation in STZ-induced diabetic mice. TBARS levels are an index of oxidative stress and endogenous lipid peroxidation which intensifies with increasing free radicals production. Therefore, in diabetic patients measurement of TBA-reactive substance levels is used to determine the level of oxidative stress. In addition, increased lipid peroxidation in diabetic mice might be due to stimulation of hepatic triglyceride synthesis [34].

## 4. Conclusion

In this study were isolated and characterized five biologically active compounds from bark of* Eysenhardtia polystachya*. The flavonoids were found to be 2′,4′-dihydroxychalcone-6′-O-*β*-d-glucopyranoside (**1**), *α*,3,2′,4′-tetrahydroxy-4-methoxy-dihydrochalcone-3′-C-*β*-glucopyranosy-6′-O-*β-* d *-*glucopyranoside (**2**), 7-hydroxy-5,8′-dimethoxy-6′*α*-l-rhamnopyranosyl-8-(3-phenyl-trans-acryloyl)-1-benzopyran-2-one (**3**), 6′7-dihydroxy-5,8-dimethoxy-8(3-phenyl-trans-acryloyl)-1-benzopyran-2-one (**4**), and 9-hydroxy-3,8-dimethoxy-4-prenylpterocarpan (**5**). The isolated compounds were evaluated for their antioxidant potentials in* in vitro* and* in vivo* assay finding that compound** 4** is the most potent antioxidant. Our data indicates that isolated** 1**–**5** from the bark of* Eysenhardtia polystachya* have an ability to reduce oxidative stress under diabetic conditions, prevent and/or delay the onset renal, pancreatic, and hepatic damage through decreasing of lipid peroxidation, antioxidant properties, and increasing radical scavenging enzymes activity, also reduce intracellular reactive oxygen species, and they consequently could alleviate complications of diabetes. In addition, the antioxidant properties of compounds** 1**–**5** are a promising strategy for ameliorating therapeutic effects by avoiding disorders in the normal redox reactions in healthy cells.

## Figures and Tables

**Table 1:** ^1^H NMR and ^13^C NMR spectral data for compounds **1–4** (*δ* in ppm, *J* in Hz).

	**1**		**2**		**3**		**4**	
	*δ*_H_	*δ*_C_	*δ*_H_	*δ*_C_	*δ*_H_	*δ*_C_	*δ*_H_	*δ*_C_
*α*	7.50 (1H, d, 15.8)	141.31	4.70, d, (1H, 7.5)	78.65	4.72, d, (1H, 7.5)	78.85	—	—
*β*	8.24 (1H, d, 15.8)	130.60	2.99, dd, (1H, 16.7, 3.0)	41.00	3.02, dd, (1H, 16.7, 3.0)	41.00	—	—
*β*	—	—	2.81, dd, (1H, 16.7, 13.2)	—	2.82, dd, (1H, 16.7, 13.2)	—	—	—
C=O	—	202.69	—	204.20	—	204.20	—	—
1	—	142.55	—	128.49	—	128.82	—	—
2	7.12 (2H, m, H-2, 6)	128.43	7.22 (1H, dt, 8.5, 2.0)	131.38	7.26 (1H, d, 2.0)	114.81	—	198.21
3		126.57	6.71 (1H, dt, 8.5, 2.0)	116.27	—	144.61	7.97, d (1H, 10.1)	143.48
4	7.46 (3H, m, H-3, 4, 5)	126.46	—	164.03	—	145.05	7.70, d (1H, 10.1)	126.71
4a	—	—	—	—	—	—	—	105.97
5		128.57	6.51 (1H, dd, 8.5, 1.9)	114.86	7.0 (1H, d, 8.0)	114.59	—	143.48
6		130.60	7.26 (1H, dd, 8.5, 2.0)	130.16	7.67 (1H, dd, 8.0, 2.0)	146.64	6.47, s	106.82
7							—	163.17
8								105.07
8a								159.82
1′	—	103.79	—	120.52	—	104.76	—	204.21
2′	6.92 (1H, s, OH-2′)	157.57	—	164.03	—	164.13	7.32, d (1H, 15.4)	142.53
3′	6.52 (1H, d, *J* = 2.4)	114.82	6.68, s	111.30	—	107.88	7.47, d (1H, 15.4)	132.26
4′	6.81 (1H, s, OH-4′)	148.36	—	144.59	—	165.34	—	132.68
5′	6.42 (1H, dd, 8.6, 2.4)	107.52	6.44 (1H, d, 8.9)	108.67	6.68, s	162.45	6.27, 1H, m	107.14
6′	—	161.21	7.71 (1H, d, 8.9)	131.78	—	164.04	—	163.56
7′							6.32 (1H, dd, 1.7, 1.7)	106.29
8′							—	166.12
9′							6.40, 1H, m	101.23
1′′	Glc-6: 5.02 (1H, d, 7.4)	106.19	Glc-2: 5.02 (1H, d, 7.3)	106.12	Glc-3: 4.85 (1H, d, 7.5)	106.07	Rham-1: 5.05, brs	100.8
2′′	3.51 (1H, m, H-2′′, 3′′)	78.36	3.53 (1H, m, H-2′′, 3′′)	78.84	3.79 (1H, t, 11.0)	74.73	3.89, m	71.50
3′′		75.59		74.02	3.55 (1H, m)	78.85	3.12, m	71.62
4′′	3.43 (1H, m, H-4′′, 5′′)	71.82	3.44 (1H, m, H-4′′, 5′′)	71.81	3.5 (1H, m)	70.27	3.12, m	72.76
5′′		71.17		71.23	3.43 (1H, m)	77.32	3.89, m	68.32
6′′	3.88 (2H, dd, 2.0, 12 H-6′′)	66.11	3.78 (2H, dd, 2.0, 12 H-6′′)	61.33	4.30 (2H, dd, 2.0, 12 H-6′′)	61.33	1.15 (3H, d, 7.0)	17.98
1′′′					Glc-6: 5.01 (1H, d, 7.3)	104.75		
2′′′					3.52 (1H, m, H-2′′′, 3′′′)	78.65		
3′′′						74.03		
4′′′					3.44 (1H, m, H-4′′′, 5′′′)	71.25		
5′′′						78.01		
6′′′					3.88 (2H, dd, 2.0, 12 H-6′′′)	62.16		
OMe					3.85, s	56.78	3.39, s, 3.46, s	60.69, 55.79
OH-4	9.10, s							
OH-2′	13.2, s							
OH-4′	6.81, s							
OH-7′							13.59, s	
OH-7							14.15, s	

**Table 2:** ^1^H NMR and ^13^C NMR spectral data for compounds **5-6** (*δ* in ppm, *J* in Hz).

	**5**		**6**	
	*δ*_H_	*δ*_C_	*δ*_H_	*δ*_C_
1	—		7.15, d (1H, 8.5)	128.72
2	—	198.16	6.52, d (1H, 8.5)	110.83
3	7.96, d (1H, 10.1)	143.48	—	147.76
4	7.71, d (1H, 10.1)	126.69	—	115.84
4a	—	105.95	—	154.59
5		143.41	—	—
6*α*	6.41, s	105.82	3.59, t (1H, 10.2)	69.37
6*β*	—	—	4.31, dd (1H, 4.9, 10.2)	69.55
6a	—	—	3.50, (1H, m, 10.2, 7.0, 5.0)	40.48
6b	—	—	—	116.56
7		168.38	6.73, s	108.07
8		105.08	—	152.51
8a		159.81	—	—
9			—	163.17
10			6.49, s	99.01
10a			—	154.59
11a			5.20, d (1H, 7.0)	77.75
11b			—	112.23
1′		204.19	3.43, d (2H, 8)	24.61
2′	7.31, d (1H, 15.4)	142.54	5.29, d (1H, 8)	121.62
3′	7.47, d (1H, 15.4)	132.26	—	137.49
4′	—	132.68	1.81, 3H, s	18.75
5′	6.26, 1H, m	108.31	1.70, 3H, s	24.61
6′	—	166.38	—	—
7′	6.32 (1H, dd, 1.7, 1.7)	105.79		
8′	—	166.12		
9′	6.40, 1H, m	101.24		
OMe-3	—	—	3.11, s	55.57
OMe-8	—	—	3.35, s	60.64
OH-9	—	—	8.12, s	—

**Table 3:** Scavenging effects of **1–5** on different *in vitro* assays.

Assay	Scavenging effects (IC_50_ *µ*g/mL)					
	**1**	**2**	**3**	**4**	**5**	Reference compounds
DPPH radical	39.39 ± 2.57	83.43 ± 5.11	12.49 ± 3.98	10.21 ± 4.23	15.45 ± 7.51	Ascorbic acid 54.14 ± 2.87 Gallic acid 3.76 ± 0.82
NO radical	48.69 ± 6.27	85.04 ± 4.83	30.29 ± 3.48	25.47 ± 4.70	31.52 ± 2.93	Ascorbic acid 31.27 ± 5.21 Quercetin 17.30 ± 1.98
O_2_^•−^	8.87 ± 1.05	18.86 ± 2.19	6.03 ± 0.39	5.76 ± 0.73	7.21 ± 3.22	Ascorbic acid 5.11 ± 0.48
Hydroxyl radical	44.13 ± 4.28	62.34 ± 3.78	37.45 ± 2.58	33.17 ± 6.83	36.52 ± 5.40	Ascorbic acid 30.29 ± 6.17
Hydrogen peroxide	91.81 ± 4.57	143.13 ± 7.55	80.73 ± 6.76	87.68 ± 9.43	101.03 ± 10.88	Ascorbic acid 443.76 ± 7.66

	Scavenging effects (%)					

Chelating activity 50 *µ*g/mL	76	43	89	84	85	Ascorbic acid 91
Peroxidationof BSA						Ascorbic acid 94
100 *µ*g/mL	30	25	36	38	32	
200 *µ*g/mL	55	48	60	64	59	
300 *µ*g/mL	83	65	90	97	92	

	TEAC (nM)					

TEAC: ABTS	28.11 ± 2.48	49.51 ± 4.15	19.38 ± 3.29	16.23 ± 2.15	20.34 ± 2.17	Curcumin 12.04 ± 4.19

The data represent the means ± SD of three determinations. IC_50_: concentration required to inhibit 50% of the activity; DPPH: 2,2-diphenyl-1-picrylhydrazyl; O_2_^•−^: superoxide radical; TEAC: ABTS (2,2′-azino-bis(3-ethylbenzothiazoline-6-sulphonic acid) radical scavenging capacity in Trolox equivalence (nanomoles); BSA: bovine serum albumin; Trolox: 6-hydroxy-2,5,7,8-tetramethylchroman-2-carboxylic acid.

**Table 4:** Effect of **1–5** on cellular oxidation stress reduction activity.

Group	DCF fluorescence (%)
Blank	0.7 ± 0.004
Control	89.2 ± 3.32
**1**	
10 *µ*g/mL	79.4 ± 5.32
50 *µ*g/mL	64.3 ± 1.83
100 *µ*g/mL	44.6 ± 4.39
**2**	
10 *µ*g/mL	86.5 ± 6.17
50 *µ*g/mL	68.4 ± 5.38
100 *µ*g/mL	55.2 ± 7.35
**3**	
10 *µ*g/mL	72.3 ± 7.09
50 *µ*g/mL	60.8 ± 4.21
100 *µ*g/mL	39.6 ± 3.19
**4**	
10 *µ*g/mL	69.8 ± 3.26
50 *µ*g/ml	52.1 ± 2.99
100 *µ*g/mL	35.1 ± 2.67
**5**	
10 *µ*g/mL	73.4 ± 5.16
50 *µ*g/mL	58.5 ± 6.43
100 *µ*g/mL	38.3 ± 4.29
Ascorbic acid 25 *μ*g/mL	19.1 ± 1.46

Fluorescence (488 nm (excitation) and 535 nm (emission)) was finally quantified after 24 h incubation. Data is shown as mean ± SD of the experiments (3 replicates each).

**Table 5:** Antioxidative status of mice and biochemical parameters at the end of the experimental period.

Organ	Group	SOD	CAT	CSH-Px	GSH	MDA
		(U/min^−1^)	(U/s^−1^)	(U/mL^−1^)	(U/mL^−1^)	(nmol/mL^−1^)
Kidney	N	2.2 ± 0.34^a^	0.74 ± 0.005^b^	68.65 ± 2.43^a^	25.27 ± 4.31^a^	29.12 ± 3.57^a^
	MD	2.8 ± 0.26^b^	0.59 ± 0.002^c^	56.41 ± 5.19^b^	5.29 ± 1.84^c^	25.60 ± 5.16^b^
	MD + **1**	3.6 ± 0.34^c^	0.79 ± 0.005^b^	71.65 ± 6.73^c^	16.21 ± 1.53^b^	27.93 ± 4.52^a^
	MD + **2**	3.1 ± 0.39^b^	0.76 ± 0.006^a^	67.62 ± 4.10^a^	13.07 ± 3.76^a^	26.62 ± 1.47^a^
	MD + **3**	3.7 ± 0.39^c^	0.82 ± 0.006^a^	73.80 ± 6.14^b^	17.23 ± 1.95^b^	28.18 ± 3.52^c^
	MD + **4**	3.8 ± 0.42^c^	0.84 ± 0.009^a^	79.37 ± 4.42^a^	19.87 ± 2.25^c^	29.14 ± 5.32^c^
	MD + **5**	3.7 ± 0.39^c^	0.82 ± 0.004^a^	74.26 ± 5.28^a^	19.01 ± 4.78^c^	28.01 ± 4.11^c^

Liver	N	6.5 ± 0.18^a^	0.82 ± 0.011^a^	99.41 ± 6.16^a^	48.76 ± 4.84^a^	26.35 ± 4.25^a^
	MD	5.8 ± 0.13^b^	0.58 ± 0.005^c^	39.34 ± 5.63^c^	26.48 ± 2.60^b^	23.78 ± 2.17^b^
	MD + **1**	6.2 ± 0.72^a^	0.71 ± 0.006^b^	79.59 ± 3.54^a^	40.63 ± 3.62^c^	25.77 ± 2.83^a^
	MD + **2**	6.0 ± 0.28^a^	0.65 ± 0.004^a^	51.24 ± 6.10^a^	35.89 ± 4.29^c^	24.21 ± 4.76^a^
	MD + **3**	6.3 ± 0.51^a^	0.73 ± 0.003^b^	90.21 ± 3.29^b^	44.21 ± 7.36^a^	26.53 ± 2.80^a^
	MD + **4**	6.4 ± 0.73^a^	0.79 ± 0.005^a^	93.26 ± 5.17^b^	47.02 ± 5.42^c^	27.91 ± 5.52^a^
	MD + **5**	6.1 ± 0.45^a^	0.76 ± 0.002^a^	89.38 ± 6.35^b^	44.32 ± 6.15^a^	27.22 ± 5.16^a^

Pancreas	N	4.03 ± 0.37^a^	0.74 ± 0.007^a^	359.56 ± 4.67^a^	51.26 ± 3.35^a^	28.56 ± 5.42^a^
	MD	2.90 ± 0.67^c^	0.65 ± 0.003^c^	325.39 ± 6.23^b^	19.48 ± 1.84^b^	24.21 ± 3.43^b^
	MD + **1**	3.70 ± 0.49^b^	0.72 ± 0.004^b^	342.29 ± 4.67^c^	43.17 ± 3.27^c^	26.55 ± 2.67^ac^
	MD + **2**	3.06 ± 0.30^a^	0.69 ± 0.005^a^	330.56 ± 7.52^b^	30.84 ± 5.63^d^	25.19 ± 6.54^a^
	MD + **3**	3.80 ± 0.59^b^	0.73 ± 0.005^b^	346.99 ± 6.43^c^	45.80 ± 3.52^c^	26.56 ± 2.90^ac^
	MD + **4**	3.89 ± 0.47^b^	0.75 ± 0.008^a^	350.48 ± 5.39^a^	47.22 ± 5.42^d^	27.84 ± 4.53^a^
	MD + **5**	3.12 ± 0.62^c^	0.76 ± 0.009^a^	349.14 ± 5.84^d^	45.74 ± 6.21^d^	27.18 ± 5.84^a^

Each value represents the mean ± SEM from 6 rats. Values within columns bearing the same lower case letters (a, b, c, and d) are not different at *p* < 0.05 and are not in any particular order. Normal control (N) and diabetic control (MD).

**Table 6:** Effects of **1–5** on serum enzyme levels in hyperglycaemic and normal mice.

Group	Glucose	SGOT	SGPT	ALP	TB	Total protein
	(mg/dL)	(IU/L)	(IU/L)	(KA units)	(mg/dL)	(g/dL)
Normal control	99.4 ± 5.67	140.18 ± 8.15	99.31 ± 4.59	30.17 ± 3.52	0.65 ± 0.07	8.1 ± 2.14
Control diabetic MD	256.7 ± 8.43^a^	234.23 ± 5.71^a^	200.06 ± 7.34^a^	71.86 ± 5.48^a^	0.46 ± 0.06^a^	5.1 ± 1.6^a^
MD + **1**	—	168.87 ± 5.15^b^	135.61 ± 6.82^b^	54.78 ± 1.98^b^	0.51 ± 0.07^b^	6.3 ± 1.84^b^
MD + **2**	—	182.04 ± 4.17^b^	145.71 ± 6.04^b^	63.62 ± 4.26^b^	0.49 ± 0.04^b^	5.9 ± 0.98^b^
MD + **3**	—	163.61 ± 7.48^b^	129.72 ± 4.48^b^	50.09 ± 3.56^b^	0.55 ± 0.05^b^	6.8 ± 2.74^b^
MD + **4**	—	150.29 ± 6.41^b^	122.27 ± 5.67^b^	45.30 ± 2.92^b^	0.58 ± 0.03^b^	7.1 ± 2.31^b^
MD + **5**	—	156.52 ± 5.42^b^	128.30 ± 7.03^b^	48.41 ± 2.68^b^	0.56 ± 0.08^b^	6.9 ± 1.95^b^

Each value represents the mean ± SEM (*n* = 6 rats). ^a^ *p* < 0.001 compared with normal control group and ^b^ *p* < 0.001 compared with MD control group. ALP: alkaline phosphatase; SGOT: serum glutamate oxaloacetate transaminase; SGPT: serum glutamate; TB: total bilirubin.

**Table 7:** Effect of **1–5 **on malondialdehyde concentration in liver and kidney of normal and diabetic mice.

Groups (mg/kg)	TBARS (*µ*M/g)	
	Liver	Kidney
Normal control	0.99 ± 0.08	1.9 ± 0.04
MD control	1.60 ± 0.09^a^	2.7 ± 0.06^a^
MD + **1**	1.07 ± 0.06^b^	2.0 ± 0.09^b^
MD + **2**	1.21 ± 0.02^b^	2.3 ± 0.07^b^
MD + **3**	0.99 ± 0.07^b^	1.9 ± 0.08^b^
MD + **4**	0.97 ± 0.03^b^	1.8 ± 0.04^b^
MD + **5**	0.95 ± 0.05^b^	1.6 ± 0.06^b^

All values are expressed as mean ± SEM, *n* = 10. ^a^ *p* < 0.05 when compared to normal control group. ^b^ *p* < 0.01 when compared to diabetic control group.

## References

[1] Jomova K., Valko M. (2011). Advances in metal-induced oxidative stress and human disease. *Toxicology*.

[2] Suntres Z. E. (2002). Role of antioxidants in paraquat toxicity. *Toxicology*.

[3] Perez R. M. G., Vargas R., Perez G. S., Zavala S. (1998). Antiurolithiatic activity of *Eysenhardtia polystachya* aqueous extract on rats. *Phytotherapy Research*.

[4] Burns D. T., Dalgarno B. G., Gargan P., Grimshaw J. (1984). An isoflavone and a coumestan from eysenhardtia polystachya-Robert Boyle's fluorescent acid-base indicator. *Phytochemistry*.

[5] Alvarez L., Rios M. Y., Esquivel C. (1998). Cytotoxic isoflavans from *Eysenhardtia polystachya*. *Journal of Natural Products*.

[6] Gutierrez R. M., Baez E. G. (2014). Evaluation of antidiabetic, antioxidant and antiglycating activities of the *Eysenhardtia polystachya*. *Pharmacognosy Magazine*.

[7] Nolis P., Parella T. (2005). Spin-edited 2D HSQC–TOCSY experiments for the measurement of homonuclear and heteronuclear coupling constants: application to carbohydrates and peptides. *Journal of Magnetic Resonance*.

[8] Chen H.-Y., Yen G.-C. (2007). Antioxidant activity and free radical-scavenging capacity of extracts from guava (*Psidium guajava* L.) leaves. *Food Chemistry*.

[9] Erel O. (2004). A novel automated direct measurement method for total antioxidant capacity using a new generation, more stable ABTS radical cation. *Clinical Biochemistry*.

[10] Marcocci L., Maguire J. J., Droylefaix M. T., Packer L. (1994). The nitric oxide-scavenging properties of *Ginkgo biloba* extract EGb 761. *Biochemical and Biophysical Research Communications*.

[11] Kumaran A., Karunakaran R. J. (2007). In vitro antioxidant activities of methanol extracts of five *Phyllanthus* species from India. *LWT-Food Science and Technology*.

[12] Halliwell B., Gutteridge J. M. C. (1990). Role of free radicals and catalytic metal ions in human disease: an overview. *Methods in Enzymology*.

[13] Ruch R. J., Cheng S. J., Klauning J. E. (1989). Prevention of citotoxicity and inhibition of intercelular communication by antioxidant catechins from Reactive oxygen Chinese Green tea. *Carcinogenesis*.

[14] Aranda A., Sequedo L., Tolosa L. (2013). Dichloro-dihydro-fluorescein diacetate (DCFH-DA) assay: A quantitative method for oxidative stress assessment of nanoparticle-treated cells. *Toxicology in Vitro*.

[15] Tahara A., Matsuyama-Yokono A., Nakano R., Someya Y., Shibasaki M. (2008). Hypoglycaemic effects of antidiabetic drugs in streptozotocin-nicotinamide-induced mildly diabetic and streptozotocin-induced severely diabetic rats. *Basic and Clinical Pharmacology and Toxicology*.

[16] Bradford M. M. (1976). A rapid and sensitive method for the quantitation of microgram quantities of protein utilizing the principle of protein-dye binding. *Analytical Biochemistry*.

[17] Fraga C. G., Leibovitz B. E., Tappel A. L. (1988). Lipid peroxidation measured as thiobarbituric acid-reactive substances in tissue slices: characterization and comparison with homogenates and microsomes. *Free Radical Biology and Medicine*.

[18] Uchiyama M., Mihara M. (1978). Determination of malonaldehyde precursor in tissues by thiobarbituric acid test. *Analytical Biochemistry*.

[19] Quadri-Spinelli T., Heilmann J., Rali T., Sticher O. (2000). Bioactive coumarin derivatives from the fern *Cyclosorus interruptus*. *Planta Medica*.

[20] Wei H., Zhang X., Wu G. (2013). Chalcone derivatives from the fern *Cyclosorus parasiticus* and their anti-proliferative activity. *Food and Chemical Toxicology*.

[21] Jiménez-González L., Álvarez-Corral M., Muñoz-Dorado M., Rodríguez-García I. (2005). A concise and diastereoselective total synthesis of *cis* and *trans*-pterocarpans. *Chemical Communications*.

[22] Maver M., Queiroz E. F., Wolfender J.-L., Hostettmann K. (2005). Flavonoids from the stem of *Eriophorum scheuchzeri*. *Journal of Natural Products*.

[23] Huang X.-Z., Bai X.-S., Liang H. (2013). Cytotoxic isoflavanones from *Uraria clarkei*. *Bulletin of the Korean Chemical Society*.

[24] Burns D. T., Dalgarno B. G., Gargan P. E., Grimshaw J. (1984). An isoflavone and a coumestan from *Eysenhardtia polystachya*-Robert Boyle's fluorescent acid-base indicator. *Phytochemistry*.

[25] Waalkens-Berendsen D. H., Kuilman-Wahls M. E. M., Bär A. (2004). Embryotoxicity and teratogenicity study with neohesperidin dihydrochalcone in rats. *Regulatory Toxicology and Pharmacology*.

[26] Roowi S., Crozier A. (2011). Flavonoids in tropical *Citrus* species. *Journal of Agricultural and Food Chemistry*.

[27] Simpson M. J., Hjelmqvist D., López-Alarcón C. (2013). Anti-peroxyl radical quality and antibacterial properties of rooibos infusions and their pure glycosylated polyphenolic constituents. *Molecules*.

[28] Innok P., Rukachaisirikul T., Phongpaichit S., Suksamrarn A. (2010). Fuscacarpans A-C, new pterocarpans from the stems of *Erythrina fusca*. *Fitoterapia*.

[29] Dai J., Mumper R. J. (2010). Plant phenolics: extraction, analysis and their antioxidant and anticancer properties. *Molecules*.

[30] Ruberto G., Baratta M. T. (2000). Antioxidant activity of selected essential oil components in two lipid model systems. *Food Chemistry*.

[31] Holst J. J. (2007). The physiology of glucagon-like peptide 1. *Physiological Reviews*.

[32] O'Brien T., Nguyen T. T., Zimmerman B. R. (1998). Hyperlipidemia and diabetes mellitus. *Mayo Clinic Proceedings*.

[33] Matkovics B. M., Kotorman M., Varga I. S., Hai D. Q., Varga C. (1998). Oxidative stress in experimental diabetes induced by streptozotocin. *Acta Physiologica Hungarica*.

[34] Pociot F., Reimers J. L., Andersen H. U. (1993). Nicotinamide—biological actions and therapeutic potential in diabetes prevention. *Diabetologia*.

